# Risk Ratio and Risk Difference Estimation in Case-cohort Studies

**DOI:** 10.2188/jea.JE20210509

**Published:** 2023-10-05

**Authors:** Hisashi Noma, Munechika Misumi, Shiro Tanaka

**Affiliations:** 1 Department of Data Science, The Institute of Statistical Mathematics, Tokyo, Japan; 2 Department of Statistics, Radiation Effects Research Foundation, Hiroshima, Japan; 3 Department of Clinical Biostatistics, Graduate School of Medicine, Kyoto University, Kyoto, Japan

**Keywords:** case-cohort study, effect measure, two-phase epidemiological design, rare disease assumption, calibration estimator

## Abstract

**Background:**

In case-cohort studies with binary outcomes, ordinary logistic regression analyses have been widely used because of their computational simplicity. However, the resultant odds ratio estimates cannot be interpreted as relative risk measures unless the event rate is low. The risk ratio and risk difference are more favorable outcome measures that are directly interpreted as effect measures without the rare disease assumption.

**Methods:**

We provide pseudo-Poisson and pseudo-normal linear regression methods for estimating risk ratios and risk differences in analyses of case-cohort studies. These multivariate regression models are fitted by weighting the inverses of sampling probabilities. Also, the precisions of the risk ratio and risk difference estimators can be improved using auxiliary variable information, specifically by adapting the calibrated or estimated weights, which are readily measured on all samples from the whole cohort. Finally, we provide computational code in R (R Foundation for Statistical Computing, Vienna, Austria) that can easily perform these methods.

**Results:**

Through numerical analyses of artificially simulated data and the National Wilms Tumor Study data, accurate risk ratio and risk difference estimates were obtained using the pseudo-Poisson and pseudo-normal linear regression methods. Also, using the auxiliary variable information from the whole cohort, precisions of these estimators were markedly improved.

**Conclusion:**

The ordinary logistic regression analyses may provide uninterpretable effect measure estimates, and the risk ratio and risk difference estimation methods are effective alternative approaches for case-cohort studies. These methods are especially recommended under situations in which the event rate is not low.

## INTRODUCTION

Epidemiologic cohort studies typically require enormous costs and effort to measure certain covariates, such as genetic and histological data. The case-cohort study has been developed as an efficient study design for reducing the difficulties involved in data collection.^1^^–^^3^ Most recent methodological research on the case-cohort study has focused on censored failure time data,^1^^,^^4^^,^^5^ but the case-cohort design was first discussed for binary outcome data,^2^^,^^3^^,^^6^^,^^7^ and there have been many practical applications of this design for binary outcomes. Although most of these studies adopted ordinary logistic regression analyses that can provide valid estimates of odds ratios,^1^ the odds ratio cannot be interpreted as an epidemiologic effect measure unless it is an approximate estimate of the risk ratio under the rare disease assumption.^8^^–^^10^ Thus, many recent publications do not recommend using the odds ratio in prospective studies; for instance, the Statistical Reporting Guidelines of the *New England Journal of Medicine* recommend avoiding the use of the odds ratio when reporting the results of clinical trials.

Besides, a marked advantage of case-cohort studies is known that the risk ratio can be directly estimated only using sampled cases and subcohort information.^6^^,^^11^ Sato^7^ provided an efficient risk ratio estimator from contingency table data, and Schouten et al^12^ proposed a multivariate analysis method using the log-linear risk model. However, these conventional methods do not use the auxiliary information in the whole cohort. Recently, Noma and Tanaka^13^ proposed using binomial regression models with log and identity links by inverse probability weighting (IPW) methods via regarding the case-cohort design as a two-phase, response-selective sampling design for estimating risk ratio and risk difference. In particular, they showed the efficiency of the IPW estimator can be improved using information on auxiliary variables by adapting the calibrated or estimated weights,^14^^–^^16^ which are readily measured on all samples from the whole cohort. However, it is well known that the binomial regression analyses involve computational difficulties, because the ranges of the regression functions cannot fall within [0, 1] due to the forms of link functions, and standard statistical packages often fail to provide estimates.^17^^,^^18^ Unfortunately, there are no other effective methods for estimating the risk ratio and risk difference under the two-phase response selective sampling framework.

Furthermore, for ordinary analyses of cohort studies, pseudo-Poisson and pseudo-normal linear regressions are widely used instead of binomial regression for estimating risk ratio and risk difference.^19^^,^^20^ Zou^19^ and Cheung^20^ showed the risk ratio and risk difference can be consistently estimated if the forms of regression functions are correct, regardless of the distribution types of outcome variables. The standard errors can also be consistently estimated using sandwich estimators.

In this article, we provide effective methods for estimating the risk ratio and risk difference in case-cohort studies based on the pseudo-Poisson and pseudo-normal linear regressions. In particular, for these multivariate regressions, we apply IPW-based methods founded on two-phase, response-selective sampling. A naïve approach is the IPW estimating equation approach using the inverse of the sampling fraction of each stratum (ie, design-based weight). In addition, the precisions of the risk ratio and risk difference estimators can be improved using auxiliary variable information, which is measured on all participants in the whole cohort, by adapting the calibrated and estimated weights. We also provide computational code for R (R Foundation for Statistical Computing, Vienna, Austria) that can easily perform these methods. We assess the performance of these methods through analyses of the National Wilms Tumor Study (NWTS) data.^21^^,^^22^

## METHODS

### The case-cohort study as a two-phase, response-selective design

We suppose a cohort that consists of *N* initially disease-free participants. The participants are classified into *J* strata on the basis of baseline covariates, and let *N_j_* be the number of subjects in each stratum ( j=1,…,J ). Suppose a stratified case-cohort study within this cohort, such that *n*_0_*_j_* ≤ *N_j_* participants are randomly sampled from the *j*th stratum, with a sampling probability *r*_0_*_j_* (called the “subcohort”). Also, *n*_1_*_j_* cases samples are randomly drawn from the incident cases in the cohort, with a probability *r*_1_*_j_*. The subcohort does not depend on specific outcomes, so it can be used as a common control for multiple different outcomes. As a special case, *J* = 1 corresponds to an unstratified case-cohort study. Expensive covariates are only measured on the selected participants, and the costs of the measurements can be greatly reduced. Formally, we can regard the sampling of the cohort from the target population as the phase-1 sampling (random sampling), and the sampling of the cases and subcohort samples from the phase-1 cohort as the phase-2 sampling^13^^–^^15^; thus, the case-cohort design can be seen as a two-phase, response-selective sampling design.^23^ The sampling scheme of the case-cohort study is essentially equivalent to that of the case-control study.^13^ Although logistic regression provides an efficient estimator of the odds ratio,^24^^,^^25^ it cannot be interpreted as a relative risk measure without a rare disease assumption,^8^^–^^10^ as noted above.

### Pseudo-Poisson and pseudo-normal linear regression in case-cohort studies

In ordinary cohort data analyses, risk ratios and risk differences can be straightforwardly estimated using the conventional binomial regression model with log and identity link functions. However, they suffer from serious computational difficulties, and the maximum likelihood estimates often cannot be obtained.^17^^,^^18^ Thus, pseudo-Poisson and pseudo-normal linear regression analyses have been widely adopted as alternative computationally stable approaches in cohort studies^19^^,^^20^; these regression analyses are originally developed for count and continuous outcomes, so we use “pseudo” for them. These estimation methods do not depend on the full-distribution assumptions of Poisson and normal distributions, and the estimators are determined by the estimating equations that only depend on the first and second moment assumptions of the outcome variables.^26^^,^^27^ In particular, when the first moment assumption (ie, the functional form of the regression function involving the link function) is correct, the regression coefficients are consistently estimated. Thus, if we formally fit the Poisson and normal linear regressions to the binomial outcome data, we can obtain consistent estimators of the regression coefficients (ie, the risk ratio and risk difference estimators). Furthermore, if the second moment assumption is incorrect, the ordinary model variance estimators are biased, but we can obtain consistent variance estimators using the sandwich variance estimators.^27^ Due to the misspecification of the second moment, the resultant estimator cannot be semiparametrically efficient, but several numerical evaluations have shown that the efficiency losses are not very large. As shown by Noma and Tanaka,^13^ binomial regression analyses can also be adopted for analyses of case-cohort studies using IPW methods; however, they involve the same computational difficulties and often cannot be implemented using the default settings of standard statistical software.

First, we introduce the notations. First, let *y* denote a binary outcome, that is *y* = 1 (for diseased subjects) and *y* = 0 (for non-diseased subjects). Also, we classify the participants in the cohort by the outcome and the *J* strata based on the baseline covariates. We denote *N_ij_* as the number of subjects in the cohort, with *y* = *i* (*i* = 0, 1) and *j*th stratum ( j=1,…,J ). In the stratified case-cohort sampling, *n*_0_*_j_* participants are sampled from *N*_0_*_j_* + *N*_1_*_j_* cases and non-cases, and *n*_1_*_j_* participants are also sampled from *N*_1_*_j_* cases. We denote the total numbers of cases and non-cases in the *n*_0_*_j_* + *n*_1_*_j_* samples as *m*_1_*_j_* and *m*_0_*_j_*, respectively. Also, for the case-cohort samples, we measure a *p*-dimensional covariate vector ***x****_ijk_* ( $k=1,\ldots ,m_{ij}$ ). We formally consider the following Poisson and normal linear regression models for the binomial outcomes:

$$\log\{E[y|x;\beta ]\}=\beta_{0}+\beta_{1}x_{1}+\ldots +\beta_{p}x_{p}$$

$$E[y|x;\beta ]=\beta_{0}+\beta_{1}x_{1}+\ldots +\beta_{p}x_{p}$$

Note that we cannot obtain consistent estimators of the regression coefficients that simply fit the pseudo-Poisson and pseudo-normal linear regression analyses for the phase-2 samples.

### IPW estimation: design weights

To provide a valid estimator of β , the IPW estimating equation approach using the inverse of the sampling fraction of each stratum is straightforwardly applied in these regression models; it corresponds to the Borgan’s type-II estimator for the Cox regression.^5^ We suppose the efficient score of the Poisson or normal linear regression as

$$U_{ijk}(\beta )=\frac{\partial \log L(\beta ;y_{ijk},x_{ijk})}{\partial \beta }$$

The IPW estimator is obtained by solving the following weighted estimating equation using the reciprocals of phase-2 sampling fractions *λ_ij_* = *N_ij_*/*m_ij_* as the weights (we denote these weights as the “design” weights):

$$\sum_{(i,j,k)\in \Xi }\lambda_{ij}U_{ijk}(\beta )=0$$

where Ξ is the index set of the phase-2 samples. Under regularity conditions, the resultant estimator is a consistent and asymptotically normal estimator of the regression coefficients as like the propensity score weighted estimator.^28^

### IPW estimation: calibrated weights

The IPW estimator with design weights is a simple and effective tool for the analysis of case-cohort studies, but it does not fully incorporate the auxiliary variable information measured on the entire cohort. Breslow et al^14^^,^^15^ proposed applying the calibration method developed in the sample survey theory^29^^,^^30^ for the IPW estimation to incorporate the auxiliary variable information. The calibration method is an adjustment method of the naïve design weights using the auxiliary variable information through a restriction known as the calibration equation, which the total of a certain variable *Q* of the entire cohort is equal to its weighted sum among the phase-2 samples.^29^^,^^30^ To achieve the most efficient estimation, Breslow et al^14^^,^^15^ proposed to use dfbetas as auxiliary variables; dfbetas are influential statistics that measure the differences of regression parameter estimates with and without the corresponding participant data. An approximate computational method of the dfbetas is the plug-in approach of Kulich and Lin.^31^ The details of the calibration method are presented in eMaterial 1. Through a weighted pseudo-Poisson or pseudo-normal linear regression analysis using the calibrated weights, we can obtain the adjusted IPW estimate. As shown in the Results section, precisions of the regression coefficient estimators are greatly improved through adapting the calibrated weights.

### IPW estimation: estimated weights

To improve the precision of the IPW estimator, an alternative approach is to adopt “estimated weight” by an adequate statistical model (ie, to substitute the weight with the reciprocal of true inclusion probability estimated from an adequate regression model that predicts whether each participant is sampled at phase-2).^14^^,^^15^^,^^32^ The estimated weights have substantial errors from the true weights (inverses of the true sampling probabilities), but the resultant weighted estimator with estimated weights is more efficient.^16^^,^^32^ When estimating the sampling probability, logistic regression is usually used. Note that it is essential to include dummy variables of the sampling strata as explanatory variables to account for the heterogeneity of sampling probabilities across the strata. In addition, the dfbetas that are used in the calibration method are recommended to be included as explanatory variables for the sake of efficiency; the dfbetas usually correlate with the phase-2 variable, and the overall model fitting is expected to be improved. Using the estimated weights, the precision of IPW estimator is also generally improved.

### Computations

All of these IPW estimators for risk ratios and risk differences can be computed by simple commands using R, especially using the survey package.^16^^,^^33^ Example code is presented in eMaterial 2, and it can be easily applied to general case-cohort datasets. Note that the computations of the variance estimators are complicated because the overall robust variance estimators of the joint estimating equations should be considered, but it can also be easily implemented using the survey package.^16^^,^^33^ The Wald-type confidence intervals of risk ratios and risk differences can also be computed using the robust variance estimates.

## RESULTS

To assess the effectiveness of the proposed methods, we conducted analyses of simulated case-cohort studies based on the NWTS Group clinical trials,^21^^,^^22^ which included 3,915 children diagnosed with Wilms’ tumor. Through the simulation studies, accuracies and precisions of the proposed estimators can be quantitatively evaluated. In the NWTS trials,^21^^,^^22^ patients with one of the rare tumor types with “unfavorable histology” (UH) had high risks of relapse and death compared with the patients whose tumors had “favorable histology” (FH). The definitive histologic diagnoses were determined at the NWTS Group Pathology Center. Although institutional pathologists used the same diagnostic procedures, they misclassified about 2% of the FH tumors as UH, and nearly 30% of the UH tumors as FH. The correct classification rates were certainly large, so the institutional diagnosis can be regarded as a surrogate variable. We simulated hypothetical case-cohort studies that treated the central histologic diagnosis as a phase-2 variable based on the NWTS cohort. Also, the outcome was “relapse within 3 years”. For the 3,915 phase-1 cohort patients, the available covariates were the institutional histological tumor type, tumor stage (coded as I–IV), age at diagnosis, and tumor diameter. We sampled all cases and 800 subcohort cases from the NWTS cohort and stratified them by the institutional histologic diagnosis (450 FH and 153 UH overall cases; 500 FH and 300 UH subcohort cases). We simulated the 10,000 hypothetical phase-2 samples and analyzed them using the proposed IPW estimators.

We used pseudo-Poisson and pseudo-normal linear regression models that included central histology (FH = 0, UH = 1), disease stage (stages I and II = 0, stages III and IV = 1), three age levels (age ≤1, age 1–4, and age >4 years), tumor diameter, and the interaction terms “central histology × age ≤1 year” and “stage × tumor diameter” as explanatory variables. For the IPW estimator with calibrated weights, we used the Poisson deviance function for calibration. For the IPW estimator with estimated weights, we calculated the weights using a logistic regression model involving the stratum indicator and the dfbetas, which were obtained by calculating the calibrated weights.

For illustrative purpose, we present the odds ratio, risk ratio, and risk difference estimates obtained from multivariate logistic, pseudo-Poisson, and pseudo-normal linear regression analyses for the NWTS dataset in Table 1. The odds ratio and risk ratio estimates were clearly different, because the event frequency was not so low (15.4%), and the odds ratio estimates could not be interpreted as accurate relative risk measures. The conventional logistic regression analyses are not suitable for such cases, and other regression models would be favorable, which provide valid estimates of interpretable epidemiologic measures. In addition, note that the binomial regression analyses with log and identity links could not provide the regression coefficients estimates for this dataset by the default settings of glm function of R.

**Table 1.** Odds ratio, risk ratio, and risk difference estimates and their 95% confidence intervals using multivariate analyses for the original NWTS dataset

	Odds ratio (Logistic regression)	Risk ratio (Pseudo-Poisson regression)	Risk difference (Pseudo-normal linear regression)
Histology	3.655 (2.876–4.646)	2.610 (2.224–3.064)	0.219 (0.170–0.268)
Stage	2.255 (1.676–3.034)	1.863 (1.487–2.318)	0.079 (0.043–0.114)
Age ≤1 year	0.747 (0.526–1.060)	0.754 (0.555–1.024)	−0.034 (−0.067–−0.002)
Age 1–4 years	0.617 (0.506–0.752)	0.677 (0.578–0.793)	−0.060 (−0.085–−0.035)
Tumor diam.	1.152 (1.081–1.228)	1.118 (1.065–1.175)	0.012 (0.006–0.019)
Hist. × Age ≤1 year	5.880 (2.961–11.674)	2.311 (1.604–3.328)	0.381 (0.263–0.498)
Stage × Tumor	0.959 (0.936–0.983)	0.969 (0.951–0.986)	−0.003 (−0.006–0.000)

NWTS, National Wilms Tumor Study.

The simulation results are presented in Table 2, including the mean, standard deviation, and root mean squared error of the estimates of 10,000 simulations and the mean of the asymptotic standard error estimates. All of the IPW estimators provided unbiased estimates from the entire cohort estimates. The efficiencies were comparable for the three IPW estimators for histologic diagnosis. Furthermore, for the other covariates that were available for all patients in the entire cohort, the efficiencies of the IPW estimators with calibrated and estimated weights were markedly gained. In particular, comparing the IPW estimators with calibrated and estimated weights, the latter generally had higher efficiency for most of the explanatory variables. In all of the 10,000 simulations, there were no failures in computations of the estimates, whereas the binomial regressions with log or identity links often failed or had convergence problems during computation of the maximum likelihood estimates. The pseudo-Poisson and pseudo-normal linear regression analyses could provide computationally stable, unbiased, and efficient estimates as like the ordinary prospective cohort analyses.

**Table 2.** NWTS data analysis using the pseudo-Poisson and pseudo-normal linear regression models, with average results from 10,000 simulated phase-2 samples based on the NWTS data (the regression coefficients are interpreted as the log risk ratio and risk difference)^*^

		Pseudo-Poisson regression				Pseudo-normal linear regression			
	
			IPW estimators				IPW estimators		
	
		Entire cohort	Design weight	Calibrated weight	Estimated weight	Entire cohort	Design weight	Calibrated weight	Estimated weight
Histology	Mean	0.960	0.958	0.967	0.961	0.219	0.220	0.220	0.220
	SD	—	0.067	0.066	0.064	—	0.021	0.021	0.021
	RMSE	—	0.067	0.066	0.064	—	0.021	0.021	0.021
	ASE	—	0.105	0.104	0.093	—	0.032	0.032	0.028
Stage	Mean	0.622	0.627	0.615	0.624	0.079	0.079	0.079	0.079
	SD	—	0.100	0.060	0.053	—	0.018	0.004	0.009
	RMSE	—	0.100	0.060	0.053	—	0.018	0.004	0.009
	ASE	—	0.149	0.128	0.116	—	0.025	0.019	0.015
Age ≤1 year	Mean	−0.283	−0.281	−0.291	−0.284	−0.034	−0.034	−0.034	−0.035
	SD	—	0.126	0.098	0.036	—	0.015	0.003	0.005
	RMSE	—	0.126	0.099	0.036	—	0.015	0.003	0.005
	ASE	—	0.201	0.187	0.178	—	0.022	0.017	0.013
Age 1–4 years	Mean	−0.390	−0.393	−0.389	−0.392	−0.060	−0.061	−0.060	−0.060
	SD	—	0.072	0.040	0.036	—	0.012	0.003	0.006
	RMSE	—	0.072	0.040	0.036	—	0.012	0.003	0.006
	ASE	—	0.108	0.090	0.080	—	0.018	0.013	0.010
Tumor diam.	Mean	0.112	0.113	0.110	0.113	0.012	0.012	0.012	0.012
	SD	—	0.021	0.014	0.011	—	0.003	0.001	0.002
	RMSE	—	0.021	0.015	0.011	—	0.003	0.001	0.002
	ASE	—	0.033	0.029	0.026	—	0.004	0.004	0.003
Hist. × Age ≤1 year	Mean	0.838	0.835	0.845	0.838	0.381	0.384	0.384	0.384
	SD	—	0.157	0.148	0.116	—	0.065	0.062	0.062
	RMSE	—	0.157	0.148	0.116	—	0.065	0.062	0.062
	ASE	—	0.241	0.238	0.222	—	0.083	0.080	0.069
Stage × Tumor	Mean	−0.032	−0.032	−0.031	−0.032	−0.003	−0.003	−0.003	−0.003
	SD	—	0.008	0.005	0.005	—	0.001	0.000	0.001
	RMSE	—	0.008	0.005	0.005	—	0.001	0.000	0.001
	ASE	—	0.012	0.011	0.010	—	0.002	0.002	0.001

ASE, mean of the estimates of asymptotic standard error in 10,000 simulations; IPW, inverse probability weighting; NWTS, National Wilms Tumor Study; RMSE, root mean squared error of the estimates from the entire cohort estimate in 10,000 simulations; SD, standard deviation.

^*^Mean, SD: Mean and SD of the estimates in 10,000 simulations.

In addition, we also implemented simulation experiments for further assessments of the proposed methods. The simulation designs and results are presented in eMaterial 3, eTable 1, and eTable 2.

## DISCUSSION

In the past few decades, case-cohort studies have been increasingly adopted in epidemiological and clinical studies due to various practical advantages compared with the nested case-control studies.^34^ Analyses of binomial outcomes often arise in such studies, and the frequencies of the outcome variables are often not rare, which is also the case in conventional cohort studies. The proposed methods in this article are likely to be effective tools in these practical situations because of their favorable properties and computational stability.

Several previous studies discussed using multiple imputations for analyses of case-cohort studies,^13^^,^^35^^–^^37^ although this paper treated only the IPW estimators. If these methods are applied to pseudo-Poisson regression or pseudo-normal linear regression, calculation of the variance estimators becomes a critical problem because the ordinary Rubin’s variance estimator is founded on the model variance estimator.^38^ The pseudo-Poisson regression and pseudo-normal linear regression must use the robust variance estimators. As with generalized estimating equation analyses with multiple imputations,^39^ the resultant variance estimators that are formally obtained by adapting the Rubin’s rule to the sandwich variance estimators might provide practically valid estimates, but rigorous theoretical discussions are needed. Furthermore, multiple imputations require distribution assumptions for the incomplete variables that are usually unknown in practice. Besides, the IPW estimators only require the missing mechanism assumptions that are completely specified by the response-selective sampling design. In terms of assuring the unbiasedness of the resultant estimators, the IPW estimators have an advantage in these applications.

Comparing the three IPW estimators, those with the calibrated and estimated weights are recommended in practice because of their relatively high efficiencies. Of these two improved estimators, the calibration method requires selection of the distance function, which influences the efficiencies of the resultant estimators. The IPW estimator with estimated weights does not have such redundancies and was found to provide more efficient estimators compared with the calibrated weights in the numerical studies. In addition, these methods can be applied for more general outcome-dependent sampling designs (eg, two-stage case-control design^40^), similarly.

In conclusion, the proposed pseudo-Poisson and pseudo-normal linear regressions are effective alternative analyses to the conventional logistic regression analyses for case-cohort studies. They provide accurate and efficient estimates of interpretable epidemiological measures and can be implemented using simple computational commands.

## SUPPLEMENTARY MATERIAL

eMaterial 1. Details of the calibration method

eMaterial 2. R example codes

eMaterial 3. Simulation experiments

eTable 1. Summary of the estimates of pseudo-Poisson regression parameters derived from 10,000 simulated datasets of a phase-2 cohort (*N* = 500) sampled from a phase-1 cohort (*N* = 4,000); the regression coefficients are interpreted as the log risk ratio^*^

eTable 2. Summary of the estimates of pseudo-normal linear regression parameters derived from 10,000 simulated datasets of a phase-2 cohort (*N* = 500) sampled from a phase-1 cohort (*N* = 4,000); the regression coefficients are interpreted as the risk difference^*^

## References

[1] Prentice RL. A case-cohort design for epidemiologic cohort studies and disease prevention trials. Biometrika. 1986;73:1–11. 10.1093/biomet/73.1.1

[2] Kupper LL, McMichael AJ, Spirtas R. A hybrid epidemiologic study design useful in estimating relative risk. J Am Stat Assoc. 1975;70:524–528. 10.2307/2285927

[3] Miettinen O. Design options in epidemiologic research: an update. Scand J Work Environ Health. 1982;8(Suppl 1):7–14. 6980462

[4] Barlow WE, Ichikawa L, Rosner D, Izumi S. Analysis of case-cohort designs. J Clin Epidemiol. 1999;52:1165–1172. 10.1016/S0895-4356(99)00102-X 10580779

[5] Borgan O, Langholz B, Samuelsen SO, Goldstein L, Pogoda J. Exposure stratified case-cohort designs. Lifetime Data Anal. 2000;6:39–58. 10.1023/A:1009661900674 10763560

[6] Sato T. Risk ratio estimation in case-cohort studies. Environ Health Perspect. 1994;102(Suppl 8):53–56. 10.1289/ehp.94102s853 7851332 PMC1566546

[7] Sato T. Maximum likelihood estimation of the risk ratio in case-cohort studies. Biometrics. 1992;48:1215–1221. 10.2307/2532713

[8] Greenland S. Interpretation and choice of effect measures in epidemiologic analysis. Am J Epidemiol. 1987;125:761–768. 10.1093/oxfordjournals.aje.a114593 3551588

[9] Sinclair JC, Bracken MB. Clinically useful measures of effect in binary analyses of randomized trials. J Clin Epidemiol. 1994;47:881–889. 10.1016/0895-4356(94)90191-0 7730891

[10] Nurminen M. To use or not to use the odds ratio in epidemiologic analyses. Eur J Epidemiol. 1995;11:365–371. 10.1007/BF01721219 8549701

[11] Rothman KJ, Greenland G, Lash TL. *Modern Epidemiology*. 3rd ed. Philadelphia: Lippincott Williams & Wilkins; 2008.

[12] Schouten EG, Dekker JM, Kok FJ, . Risk ratio and rate ratio estimation in case-cohort designs: hypertension and cardiovascular mortality. Stat Med. 1993;12:1733–1745. 10.1002/sim.4780121808 8248665

[13] Noma H, Tanaka S. Analysis of case-cohort designs with binary outcomes: improving the efficiency using whole cohort auxiliary information. Stat Methods Med Res. 2017;26:691–706. 10.1177/0962280214556175 25348675

[14] Breslow NE, Lumley T, Ballantyne CM, Chambless LE, Kulich M. Using the whole cohort in the analysis of case-cohort data. Am J Epidemiol. 2009;169:1398–1405. 10.1093/aje/kwp055 19357328 PMC2768499

[15] Breslow NE, Lumley T, Ballantyne CM, Chambless LE, Kulich M. Improved Horvitz-Thompson estimation of model parameters from two-phases stratified samples: applications in epidemiology. Stat Biosci. 2009;1:32–49. 10.1007/s12561-009-9001-6 20174455 PMC2822363

[16] Lumley T, Shaw PA, Dai JY. Connections between survey calibration estimators and semiparametric models for incomplete data. Int Stat Rev. 2011;79:200–220. 10.1111/j.1751-5823.2011.00138.x 23833390 PMC3699889

[17] McNutt LA, Wu C, Xue X, Hafner JP. Estimating the relative risk in cohort studies and clinical trials of common outcomes. Am J Epidemiol. 2003;157:940–943. 10.1093/aje/kwg074 12746247

[18] Wallenstein S, Bodian C. Epidemiologic programs for computers and calculators. Inferences on odds ratios, relative risks, and risk differences based on standard regression programs. Am J Epidemiol. 1987;126:346–355. 10.1093/aje/126.2.346 3605061

[19] Zou G. A modified Poisson regression approach to prospective studies with binary data. Am J Epidemiol. 2004;159:702–706. 10.1093/aje/kwh090 15033648

[20] Cheung YB. A modified least-squares regression approach to the estimation of risk difference. Am J Epidemiol. 2007;166:1337–1344. 10.1093/aje/kwm223 18000021

[21] D’Angio GJ, Breslow N, Beckwith JB, . Treatment of Wilms’tumor. Cancer. 1989;64:349–360. 10.1002/1097-0142(19890715)64:2<349::AID-CNCR2820640202>3.0.CO;2-Q 2544249

[22] Green DM, Breslow NE, Beckwith JB, . Comparison between single-dose and divided-dose administration of dactinomycin and doxorubicin for patients with Wilms tumor: a report from the National Wilms Tumor Study Group. J Clin Oncol. 1998;16:237–245. 10.1200/JCO.1998.16.1.237 9440748

[23] Lawless JF, Kalbfleisch JD, Wild CJ. Semiparametric methods for response-selective and missing data problems. J R Stat Soc B. 1999;61:413–438. 10.1111/1467-9868.00185

[24] Prentice RL, Pyke R. Logistic disease incidence models and case-control studies. Biometrika. 1979;66:403–411. 10.1093/biomet/66.3.403

[25] Breslow NE, Robins JM, Wellner JA. On the semi-parametric efficiency of logistic regression under case-control sampling. Bernoulli. 2000;6:447–455. 10.2307/3318670

[26] Agresti A. *Categorical Data Analysis*. 3rd ed. Hoboken: Wiley; 2013.

[27] Tsiatis AA. *Semiparametric Theory and Missing Data*. New York: Springer; 2006.

[28] Austin PC. An introduction to propensity score methods for reducing the effects of confounding in observational studies. Multivariate Behav Res. 2011;46:399–424. 10.1080/00273171.2011.568786 21818162 PMC3144483

[29] Deville JC, Särndal CE. Calibration estimators in survey sampling. J Am Stat Assoc. 1992;87:376–382. 10.1080/01621459.1992.10475217

[30] Deville JC, Särndal CE, Sautory O. Generalized raking procedures in survey sampling. J Am Stat Assoc. 1993;88:1013–1020. 10.1080/01621459.1993.10476369

[31] Kulich M, Lin DY. Improving the efficiency of relative-risk estimation in case-control studies. J Am Stat Assoc. 2004;99:832–844. 10.1198/016214504000000584

[32] Robins JM, Rotnitzky A, Zhao LP. Estimation of regression-coefficients when some regressors are not always observed. J Am Stat Assoc. 1994;89:846–866. 10.1080/01621459.1994.10476818

[33] Lumley T. Analysis of complex survey samples. J Stat Softw. 2004;9(8):1–19. 10.18637/jss.v009.i08

[34] Thomas DC. Addendum to a paper by Liddell FDK, McDolad JC, Thomas DC, Cunliffe SV. *J Royal Stat Soc A*. 1977;140:483–485.

[35] Marti H, Chavance M. Multiple imputation analysis of case-cohort studies. Stat Med. 2011;30:1595–1607. 10.1002/sim.4130 21351290

[36] Keogh RH, White IR. Using full-cohort data in nested case-control and case-cohort studies by multiple imputation. Stat Med. 2013;32:4021–4043. 10.1002/sim.5818 23613433

[37] Keogh RH. Multiple imputation for sampled cohort data. In: Borgan Ø, Breslow NE, Chatterjee N, Gail MH, Scott A, Wild CJ, editors. *Handbook of Statistical Methods for Case-Control Studies*. Boca Raton: CRC Press; 2018:373–390.

[38] Little RJ, Rubin DB. *Statistical Analysis with Missing Data*. 2nd ed. New York: Wiley; 2002.

[39] Aloisio KM, Swanson SA, Micali N, Field A, Horton NJ. Analysis of partially observed clustered data using generalized estimating equations and multiple imputation. Stata J. 2014;14:863–883. 10.1177/1536867X1401400410 25642154 PMC4306281

[40] White JE. A two stage design for the study of the relationship between a rare exposure and a rare disease. Am J Epidemiol. 1982;115:119–128. 10.1093/oxfordjournals.aje.a113266 7055123

