# Supplementary material for: Risk Ratio and Risk Difference Estimation in Case-cohort Studies
Source: J Epidemiol. 2023 Oct 5;33(10):508–13. doi: 10.2188/jea.JE20210509 (PMC10483099; doi:10.2188/jea.JE20210509)
Supplement: Supplementary file 1 [file je-33-508-s001.pdf]

eMaterial 1. Details of the calibration method

The calibration equation used to adjust the weights of IPW estimator is provided as

$$\hat{Q}_{tot} = \sum_{(i,j,k)\in\Xi} \omega_{ijk} Q_{ijk} = \sum_{(i,j,k)\in\Omega} Q_{ijk} = Q_{tot}$$

where Q_{ijk} are the auxiliary variables that are measured for all subjects in the whole cohort, and the weights ω_{ijk} are set so that the weighted mean of Q_{ijk} is equal to the population total Q_{tot} ($i = 0,1; j = 1,\ldots,J; k = 1,\ldots,N_{ij}$); Ω is the index set of the phase-1 samples. When the target measure is the population total t_{tot} of a variable t_{ijk}, and if t_{ijk} and Q_{ijk} are strongly correlated, the Horvitz-Thompson estimator $\hat{t}_{HT} = \sum_{(i,j,k)\in\xi} \lambda_{ijk} t_{ijk}$ is improved by substituting the weights λ_{ijk} for the weights of the calibration equation ω_{ijk}. The adjusted weights $\omega_{ijk} = \gamma_{ijk}\lambda_{ijk}$ are called the "calibrated" weights; γ_{ijk} is the adjustment coefficient. Since the calibration equation does not uniquely specify the weights, the calibrated weights are defined as those nearest to the design weight λ_{ijk} based on a certain distance function $G(\omega,\lambda)$, e.g., $G_1(\omega,\lambda) = (\omega-\lambda)^2/2\omega$ (linear function) and $G_2(\omega,\lambda) = \omega\log(\omega/\lambda) - \omega + \lambda$ (Poisson deviance). See Deville and Särndal (1992) and Deville et al. (1993) for more distance functions and their properties.

In case-cohort studies using the pseudo-Poisson and pseudo-normal linear regressions, the IPW estimator with design weights is approximated to the true regression coefficient $\boldsymbol{\beta}_0$ plus a weighted sum of the efficient scores:

$$\widehat{\boldsymbol{\beta}} \approx \boldsymbol{\beta}_0 + \sum_{(i,j,k)\in\Xi} \lambda_{ijk} I_{ijk}^{-1}(\boldsymbol{\beta}_0) U_{ijk}(\boldsymbol{\beta}_0)$$

where $I_{ijk}(\boldsymbol{\beta}_0)$ is the information matrix of the Poisson or normal linear regression model. Since $\boldsymbol{\beta}_0$ is a fixed quantity, the estimator is expected to be improved by calibrating the weight with respect to some auxiliary variables correlated with $I_{ijk}^{-1}(\boldsymbol{\beta}_0)U_{ijk}(\boldsymbol{\beta}_0)$. Breslow et al. (2009a, b) proposed to use dfbetas $I_{ijk}^{-1}(\widetilde{\boldsymbol{\beta}})U_{ijk}(\widetilde{\boldsymbol{\beta}})$, where $\widetilde{\boldsymbol{\beta}}$ is the regression coefficient estimate for phase-1 cohort data. For the

computations of the dfbetas, since $\tilde{\boldsymbol{\beta}}$ is unknown, we propose using the following approximate computational method.

(i) Because the phase-2 variables are missing, Breslow et al. (2009a, b) proposed imputing a single suitable value to the missing covariates. For predicting the missing covariates, construct a regression model with the fully observed covariates as explanatory variables and make a prediction model using a weighted estimation.

(ii) For the imputed phase-1 dataset, use the predicted values generated in Step (i) to estimate $\tilde{\boldsymbol{\beta}}$ by fitting the pseudo-Poisson or pseudo-normal linear regression. Then, extract the dfbetas from the regression model.

Then, approximate dfbetas are computed using the estimates of $\tilde{\boldsymbol{\beta}}$ and the design weights are calibrated using the computed dfbetas as the auxiliary variables. We also propose to use the calibrated weights with the approximate dfbetas. Through a weighted pseudo-Poisson or pseudo-normal linear regression analysis with the calibrated weights, we can obtain the adjusted IPW estimate.

eMaterial 2. R example codes

```
library(survey)
ex1 <- read.csv("exdata.csv")    # Read the example dataset

# Please download the example dataset from:
https://www.ism.ac.jp/~noma/casecohort2021/exdata.csv
# y: Binomial outcome variable (=0, 1)
# z: Phase-2 variable that is only measured for
participants sampled at phase-2 sampling
# x1, x2, x3, u1: Explanatory variables measured for all
participants in the entire cohort
# tls: An indicator variable that divides the population by
tertiles of u1
# sam: An indicator variable that specifies whether the
corresponding participant is sampled at the phase-2
sampling

N <- dim(ex1)[1]

# IPW estimation; design weights
dccs2 <- twophase(id=list(~id, ~id), subset=~sam,
strata=list(NULL, ~interaction(tls, x3, y)), data=ex1)
  # specifying the second phase sampling design
std1 <- svyglm(y ~ z + x1 + x2 + x1*z,
family=poisson(link="log"), design=dccs2)
summary(std1)     # RR estimates; pseudo-Poisson regression
std2 <- svyglm(y ~ z + x1 + x2 + x1*z,
family=gaussian(link="identity"), design=dccs2)
summary(std2)     # RD estimates; pseudo-normal linear
regression
```

```
# IPW estimation; calibrated weights
Hmodel <- svyglm(z ~ u1 + x3, family=quasibinomial,
design=dccs2)
  # specifying the prediction model of z
ex1$estH <-
predict(Hmodel,type="response",newdata=ex1)[1:N]
calmodel1 <- glm(y ~ estH + x1 + x1*estH,
family=poisson(link="log"), data=ex1)
calmodel2 <- glm(y ~ estH + x1 + x1*estH,
family=gaussian(link="identity"), data=ex1)

db1 <- dfbetas(calmodel1) + 1
colnames(db1) <- paste("db1",1:ncol(db1),sep="")
db2 <- dfbetas(calmodel2) + 1
colnames(db2) <- paste("db2",1:ncol(db2),sep="")
ex2 <- cbind(ex1, db1, db2)

dstrt <- twophase(id=list(~id,~id),strata=list(NULL,
~interaction(tls, x3, y)),subset=~sam,data=ex2)
  # specifying the second phase sampling design
dcal1 <- calibrate(dstrt,
formula=make.formula(colnames(db1)),
pop=c(`(Intercept)`=N,colSums(db1)),calfun="raking")
dcal2 <- calibrate(dstrt,
formula=make.formula(colnames(db2)),
pop=c(`(Intercept)`=N,colSums(db2)),calfun="raking")

calib1 <- svyglm(y ~ z + x1 + x2 + x1*z,
family=poisson(link="log"), design=dcal1)
summary(calib1)         # RR estimates; pseudo-Poisson
regression
```

```
calib2 <- svyglm(y ~ z + x1 + x2 + x1*z,
family=gaussian(link="identity"), design=dcal2)
summary(calib2)         # RD estimates; pseudo-normal
linear regression

# IPW estimation; estimated weights
drrz1 <- estWeights(data=ex2,formula=~interaction(tls, x3,
y) + db11 + db12 + db13 + db14, subset=I(ex2$sam))
drrz2 <- estWeights(data=ex2,formula=~interaction(tls, x3,
y) + db21 + db22 + db23 + db24, subset=I(ex2$sam))

rrz1 <- svyglm(y ~ z + x1 + x2 + x1*z,
family=poisson(link="log"), design=drrz1)
summary(rrz1)     # RR estimates; pseudo-Poisson regression
rrz2 <- svyglm(y ~ z + x1 + x2 + x1*z,
family=gaussian(link="identity"), design=drrz2)
summary(rrz2)     # RD estimates; pseudo-normal linear
regression
```

eMaterial 3. Simulation experiments

To assess the performances of the estimation methods, we conducted simulation studies. Simulated case-cohort data were artificially generated using the two-phase stratified sampling design. The settings were the same as those in the Wilms' tumor studies presented below. For the event occurrence mechanisms, we considered the binomial regression model with a log or identity link function; $\log\{\Pr(Y = 1 \mid x_1, x_2, x_3)\} = \beta_0 + \beta_1 x_1 + \beta_2 x_2 + \beta_3 x_3 + \delta_{12} x_1 x_2$ or $\Pr(Y = 1 \mid x_1, x_2, x_3) = \beta_0 + \beta_1 x_1 + \beta_2 x_2 + \beta_3 x_3 + \delta_{12} x_1 x_2$. x_1 was supposed to be measured only for the phase-2 samples and was considered to correlate with two other variables, $z_1 \sim$ Bernoulli (0.10) and $z_2 \sim N(0, 1)$. These variables were observed for all subjects in the phase-1 cohort, such that $\text{logit}\{\Pr(X_1 = 1 \mid z_1, z_2)\} = \gamma_0 + \gamma_1 z_1 + \gamma_2 z_2$. x_2 and x_3 were supposed to be measured for all participants in a phase-1 cohort and were dummy variables of a trinomial distribution with event probabilities 0.16 and 0.48, respectively. The stratified phase-2 sampling was implemented by six strata divided by z_1 and tertiles of z_2. The sample size of the phase-1 cohort was set to 4,000. In the phase-2 sampling, all cases were sampled, and the number of cases in the subcohort was set to 400 for three strata with $z_1 = 0$ and to 100 for three strata with $z_2 = 1$. For the log link model, the regression parameters were set to $\beta_0 = -1.81$, $\beta_1 = 0.96$, $\beta_2 = -0.28$, $\beta_3 = -0.39$, and $\delta_{12} = 1.84$. Also, for the identity link model, the regression parameters were set to $\beta_0 = 0.17$, $\beta_1 = 0.22$, $\beta_2 = -0.03$, $\beta_3 = -0.06$, and $\delta_{12} = 0.38$. For the model used to generate x_1, we considered z_1 as a correlated surrogate variable of x_1. Two settings for the correlation of x_1 and z_1 were considered: (i) $\gamma_0 = -3.50$, $\gamma_1 = 0.50$, and $\gamma_2 = 4.5$ (sensitivity = 0.720, specificity = 0.967; high correlation); and (ii) $\gamma_0 = -3.50$, $\gamma_1 = 0.50$, and $\gamma_2 = 3.0$ (sensitivity = 0.384, specificity = 0.967; moderate correlation). We performed 10,000 simulations for each scenario. We compared the performance of the IPW estimators (with the design weight, calibrated weight, and estimated weight) with the entire cohort estimator as a benchmark. For the calibrated weights, we computed approximate dfbetas, predicted the missing x_1 using the logistic model $\text{logit}\{\Pr(X_1 = 1 \mid z_1, z_2)\} = \gamma_0 + \gamma_1 z_1 + \gamma_2$

z_2 (correct model), and adopted a raking distance function. For the estimated weights, we used a logistic regression model and adopted the stratum indicators and the dfbetas as covariates.

The results of the simulation studies are presented in e-Tables 1 and 2. We assessed the mean, standard deviation (SD), root mean squared error (RMSE), empirical coverage probability (CP) for the 95% confidence intervals, and estimated relative efficiency (RE) of the IPW estimators compared with the entire cohort estimator for the 10,000 simulations. All of the IPW estimators could estimate the risk ratios and risk differences without bias. The relative efficiencies depended on the scenarios and regression coefficients, but the efficiencies of the IPW estimators with calibrated and estimated weights were generally higher than the IPW estimator with design weights. In particular, efficiencies were markedly gained for the regression coefficients (β_2 and δ_{12}) that corresponded to covariates that correlated with the phase-2 variable x_1. Also, the relative efficiencies of estimating β_1 by the IPW estimators with calibrated and estimated weights were slightly improved comparing with the IPW estimator with design weights. In addition, the 95% confidence intervals were validly constricted; the CPs of all of the proposed IPW methods were approximately 0.95.

eTable 1. Summary of the estimates of pseudo-Poisson regression parameters derived from 10,000 simulated datasets of a phase-2 cohort (*N*=500) sampled from a phase-1 cohort (*N*=4,000); the regression coefficients are interpreted as the log risk ratio*

	Strong correlation					Moderate correlation				
	Mean	SE	RMSE	CP	RE	Mean	SE	RMSE	CP	RE
β_1 = 0.96										
Entire cohort	0.958	0.088	0.088	0.949	1.000	0.957	0.101	0.101	0.951	1.000
IPW (design weight)	0.958	0.097	0.097	0.949	0.821	0.958	0.116	0.116	0.949	0.756
IPW (calibrated weight)	0.959	0.097	0.097	0.948	0.823	0.958	0.116	0.116	0.948	0.757
IPW (estimated weight)	0.959	0.097	0.097	0.949	0.826	0.958	0.116	0.116	0.949	0.758
β_2 = −0.28										
Entire cohort	−0.284	0.127	0.127	0.949	1.000	−0.284	0.126	0.126	0.949	1.000
IPW (design weight)	−0.284	0.142	0.142	0.949	0.796	−0.284	0.141	0.141	0.950	0.797
IPW (calibrated weight)	−0.286	0.135	0.135	0.958	0.883	−0.286	0.133	0.133	0.961	0.889
IPW (estimated weight)	−0.285	0.129	0.129	0.959	0.961	−0.285	0.128	0.128	0.959	0.959
β_3 = −0.39										
Entire cohort	−0.390	0.079	0.079	0.955	1.000	−0.390	0.081	0.081	0.954	1.000
IPW (design weight)	−0.390	0.088	0.088	0.950	0.797	−0.391	0.091	0.091	0.951	0.792
IPW (calibrated weight)	−0.390	0.088	0.088	0.949	0.797	−0.391	0.091	0.091	0.951	0.791
IPW (estimated weight)	−0.390	0.088	0.088	0.949	0.797	−0.391	0.091	0.091	0.951	0.792
δ_{12} = 0.84										
Entire cohort	0.845	0.162	0.162	0.950	1.000	0.845	0.175	0.175	0.951	1.000
IPW (design weight)	0.845	0.186	0.186	0.949	0.754	0.846	0.208	0.208	0.946	0.712
IPW (calibrated weight)	0.846	0.179	0.180	0.957	0.811	0.847	0.204	0.204	0.952	0.738
IPW (estimated weight)	0.846	0.176	0.177	0.955	0.838	0.847	0.202	0.202	0.948	0.753

CP, empirical coverage probability for the 95% confidence intervals; RE, estimated relative efficiency of the estimator compared with the entire cohort; RMSE, root mean squared error of the estimates from the true regression parameter.

* Mean, SE: Mean and SD of the estimates in 10,000 simulations.

eTable 2. Summary of the estimates of pseudo-normal linear regression parameters derived from 10,000 simulated datasets of a phase-2 cohort (*N*=500) sampled from a phase-1 cohort (*N*=4,000); the regression coefficients are interpreted as the risk difference*

	Strong correlation					Moderate correlation				
	Mean	SE	RMSE	CP	RE	Mean	SE	RMSE	CP	RE
$\beta_1 = 0.22$										
Entire cohort	0.220	0.027	0.027	0.950	1.000	0.220	0.032	0.032	0.948	1.000
IPW (design weight)	0.220	0.030	0.030	0.948	0.797	0.221	0.038	0.038	0.946	0.729
IPW (calibrated weight)	0.220	0.030	0.030	0.948	0.800	0.221	0.037	0.037	0.946	0.732
IPW (estimated weight)	0.220	0.030	0.030	0.947	0.799	0.221	0.037	0.037	0.945	0.731
$\beta_2 = -0.03$										
Entire cohort	−0.030	0.018	0.018	0.949	1.000	−0.030	0.017	0.017	0.951	1.000
IPW (design weight)	−0.030	0.020	0.020	0.949	0.771	−0.030	0.020	0.020	0.948	0.772
IPW (calibrated weight)	−0.030	0.018	0.018	0.967	0.935	−0.030	0.018	0.018	0.970	0.935
IPW (estimated weight)	−0.030	0.018	0.018	0.950	0.935	−0.030	0.018	0.018	0.952	0.934
$\beta_3 = -0.06$										
Entire cohort	−0.060	0.013	0.013	0.953	1.000	−0.060	0.012	0.012	0.951	1.000
IPW (design weight)	−0.060	0.014	0.014	0.950	0.781	−0.060	0.014	0.014	0.951	0.775
IPW (calibrated weight)	−0.060	0.014	0.014	0.950	0.782	−0.060	0.014	0.014	0.952	0.776
IPW (estimated weight)	−0.060	0.014	0.014	0.950	0.782	−0.060	0.014	0.014	0.952	0.776
$\delta_{12} = 0.38$										
Entire cohort	0.380	0.063	0.063	0.943	1.000	0.380	0.075	0.075	0.944	1.000
IPW (design weight)	0.381	0.078	0.078	0.931	0.662	0.383	0.097	0.097	0.919	0.597
IPW (calibrated weight)	0.381	0.076	0.076	0.939	0.697	0.383	0.096	0.096	0.921	0.607
IPW (estimated weight)	0.381	0.076	0.076	0.932	0.693	0.383	0.097	0.097	0.920	0.604

CP, empirical coverage probability for the 95% confidence intervals; RE, estimated relative efficiency of the estimator compared with the entire cohort; RMSE, root mean squared error of the estimates from the true regression parameter.

* Mean, SE: Mean and SD of the estimates in 10,000 simulations.

REFERENCES

Breslow, N. E., Lumley, T., Ballantyne, C. M., Chambless, L. E., and Kulich, M. (2009a). Improved Horvitz-Thompson estimation of model parameters from two-phases stratified samples: Applications in epidemiology. *Statistics in Bioscience* **1**, 32-49.

Breslow, N. E., Lumley, T., Ballantyne, C. M., Chambless, L. E., and Kulich, M. (2009b). Using the whole cohort in the analysis of case-cohort data. *American Journal of Epidemiology* **169**, 1398-1405.

Deville, J. C., and Särndal, C.-E. (1992). Calibration estimators in survey sampling. *Journal of the American Statistical Association* **87**, 376-382.

Deville, J. C., Särndal, C.-E., and Sautory, O. (1993). Generalized raking procedures in survey sampling. *Journal of the American Statistical Association* **88**, 1013-1020.
